# Immunohistochemical detection of *Treponema pallidum* in skin samples with clinical and histopathological correlations and Warthin-Starry staining critical analysis^☆^

**DOI:** 10.1016/j.abd.2022.02.008

**Published:** 2023-03-09

**Authors:** Mariana Freitas de Assis Pereira Rosa, Leonardo Pereira Quintella, Luiz Claudio Ferreira, Tullia Cuzzi

**Affiliations:** a Postgraduate Program in Anatomical Pathology, Faculty of Medicine, Universidade Federal do Rio de Janeiro, Rio de Janeiro, RJ, Brazil; b Anatomical Pathology Service, National Institute of Infectious Diseases, Fundação Oswaldo Cruz, Rio de Janeiro, RJ, Brazil; c Department of Pathology, Faculty of Medicine, Universidade Federal do Rio de Janeiro, Rio de Janeiro, RJ, Brazil

**Keywords:** Diagnosis, Histology, Immunohistochemistry, Syphilis, *Treponema pallidum*

## Abstract

**Background:**

Syphilis in its different phases may be a difficult diagnosis in clinical and histopathological grounds.

**Objectives:**

The present study objectives were to evaluate the detection and tissue distribution of *Treponema pallidum* in skin lesions of syphilis.

**Methods:**

A blinded diagnostic accuracy study was performed with immunohistochemistry and Warthin-Starry silver staining in skin samples from patients with syphilis and other diseases. Patients attended two tertiary hospitals between 2000 and 2019. Prevalence ratios (PR) and 95% confidence intervals (95% CI) were calculated for the association between immunohistochemistry positivity and clinical-histopathological variables.

**Results:**

Thirty-eight patients with syphilis and their 40 biopsy specimens were included in the study. Thirty-six skin samples were used as non-syphilis controls. The Warthin-Starry technique was unable to accurately demonstrate bacteria in all samples. Immunohistochemistry showed spirochetes only in skin samples from patients with syphilis (24/40) with 60% sensitivity (95% CI 44.8‒75.2). Specificity was 100% and accuracy, 78.9% (95% CI 69.8‒88.1). Most cases had spirochetes in both dermis and epidermis and there was a high bacterial load.

**Study limitations:**

Correlation between immunohistochemistry and clinical or histopathological characteristics was observed but was limited statistically due to the small sample size.

**Conclusions:**

Spirochetes were promptly seen in an immunohistochemistry protocol, which can contribute to the diagnosis of syphilis in skin biopsy samples. On the other hand, the Warthin-Starry technique showed to be of no practical value.

## Introduction

Syphilis is a curable sexually transmitted infection (STI) re-emerging globally in recent years^1^, and a major public health challenge, particularly in groups with high-risk sexual behavior. In Brazil, the frequency of acquired syphilis has increased progressively over the past 10 years. This increase was as high as 30.2% in one single year, between 2017 and 2018. In the period 2010-2018, the rate of detection of syphilis in pregnant women increased from 3.5 to 21.5 cases per thousand live births^2^, ^3^.

The disease is caused by the non-cultivable spirochete bacterium *Treponema pallidum* subsp. *pallidum*. Diagnosis requires a correlation between clinical data, including previous infections and recent exposure, and laboratory tests. The most useful diagnostic method for syphilis is serological testing. Nevertheless, considering protean clinical manifestations, histopathological examination is very important when syphilis is not initially suspected.

*Treponema pallidum* is classically reported to be identified by silver staining techniques such as Warthin-Starry, Levaditi, or Dieterle modified by Steiner. However, unspecific or artefactual background staining of tissue elements, such as reticular fibers and melanin, pose interpretation difficulties which may result in false positive or false negative results^4^, ^5^, ^6^, ^7^. Recently, immunoperoxidase labeling using the polyclonal anti-*Treponema pallidum* antibody has been shown to be a sensitive and useful method to highlight and identify the spirochete^6^, ^7^, ^8^, ^9^, ^10^, ^11^. This study was carried out to assess the contribution of immunohistochemistry (IHC) and Warthin-Starry (WS) technique in the observation of spirochetes in skin biopsy samples. The correlation of this detection with clinical and histopathological findings was also investigated in order to define expected performance in different settings.

## Methods

A diagnostic accuracy study was conducted using immunohistochemical and WS staining for the detection of spirochetes in skin biopsy samples. Clinical and serological data of patients seen in two tertiary hospitals in Rio de Janeiro between 2000 and 2019 were retrieved from medical records. Patients were classified as having syphilis when clinical features were consistent and serological test was positive (Venereal Disease Research Laboratory test ‒ VDRL, with titers greater than 1/8 or positive treponemal test), or VDRL was reactive in the cerebrospinal fluid in any titration at the time of skin biopsy and diagnostic investigation. Patients with one or more skin biopsy samples were included. A control group was formed by skin samples from patients with non-reactive serological tests for syphilis and diagnosed with other dermatoses that might be considered in syphilis clinical and histopathological differential diagnosis.

Histological 4 μm thick sections were obtained from paraffin blocks for hematoxylin and eosin and WS staining according to Luna^12^. Histopathological reports were reviewed, and slides were evaluated. Inflammatory reaction patterns, infiltrate distribution, endothelial hyperplasia, and semi-quantification of plasma cells were considered in the evaluation of skin samples from patients with syphilis. Plasma cells were classified as ++/++ when they composed more than 30% of infiltrate; +/++ when less than 30%; and zero when they were absent or scarce.

Additional histological sections were obtained for immunohistochemical study performed with heat-induced epitope retrieval pre-treatment, polyclonal primary antibody against *T. pallidum* (BIOCARE Medical, California, USA) in 1:200 dilution and polymeric labeling detection system (Novolink Max Polymer Detection System, Leica Biosystems, Illinois, USA). Immunohistochemical analysis was blinded to the diagnosis and made simultaneously and consensually by two pathologists with a large experience in the histopathological and immunohistochemical diagnosis of infectious diseases, in a Nikon dual-head E200 optical microscope. The analysis of the bacterial load was made using an arbitrary semi-quantitative scale considering (3+) numerous bacteria in all fields; (2+) numerous bacteria in some fields; (1+) few bacteria.

Statistical analyses were performed using Statistical Package for the Social Sciences Software (SPSS) version 20. Prevalence Ratios (PR) and respective 95% Confidence Intervals (95% CI) were calculated to measure the association between immunohistochemistry positivity and clinical-histopathological variables. Chi-Square (χ^2^) and Fisher exact tests were used to determine statistical significance. A significance level of 0.05 was defined.

## Results

Thirty-eight patients (29 men and 9 women) ranging from 19 to 66 years old (mean: 42 years, median 39 years) with syphilis and their 40 skin specimens were included in the study. Anti-HIV serology was positive in 22 patients; 10 patients had a viral load greater than 1.000 copies/mL; 7 patients had a CD4 count less than 200 cells/mm^3^, and 6 patients had both. Skin lesions were predominant on the upper limbs (32/38). Twenty-six patients presented papules and 22 plaque lesions. Other skin lesions described were exanthema (10/38), nodules (10/38), and pustules or crusts (8/38). Twenty-nine samples (72.5%) came from patients with secondary syphilis.

**Figure 1:**
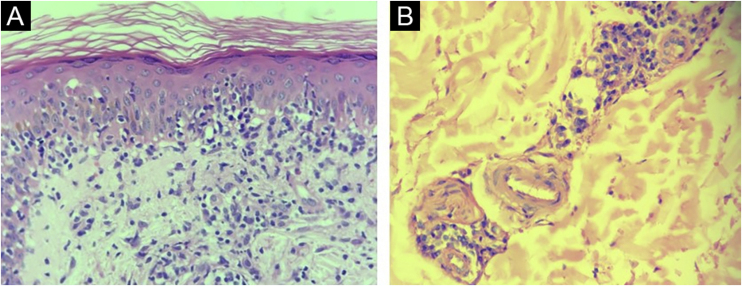
Skin, histopathological findings. (A) Interface dermatitis (Hematoxylin & eosin, ×400). **(**B) Endothelial hyperplasia and abundant plasma cells around dermal vessels (Hematoxylin & eosin, ×400).

Histopathological reports of 30/40 (75%) samples from syphilis patients were considered compatible with the diagnosis. Drug eruption, psoriasis, Reiter's syndrome, and xanthogranuloma were additional suggested histopathological diagnoses. In 5 reports (12.5%) no histopathological diagnosis suggestion was made. The most common tissue reactions were interface, superficial, and deep perivascular dermatitis (27.5%) and the most common finding was endothelial hyperplasia (75%) (Fig. 1).

Thirty-six skin samples formed a control group and were from patients diagnosed with other dermatoses (10 drug eruptions, 10 psoriasis, 4 lupus erythematosus, 4 eczemas, 2 leprosy, 2 erythrodermas, 2 vasculitis, 1 Sweet syndrome, and 1 lichen planus).

**Figure 2:**
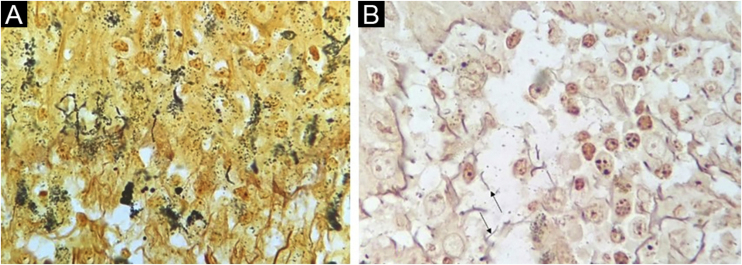
(A) Basal cell layer background staining (Warthin-Starry staining, ×1000). (B) Unspecified structures in dermis (arrows, Warthin-Starry staining, ×1000).

The WS staining was negative or inconclusive in all samples. Background staining of unspecified structures and melanin in the melanocyte dendrites made it impossible to definitely identify spirochetes (Fig. 2).

**Figure 3:**
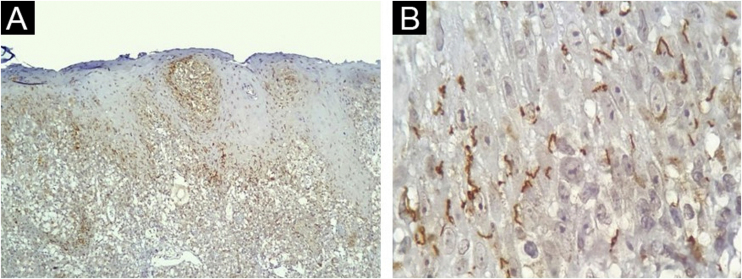
Skin, immunohistochemical findings. (A) Spirochetes in the dermal-epidermal junction and papillary dermis, (Immunohistochemistry with anti-*Treponema pallidum* antibody ×100). (B) Detail of spirochetes location in the lower epidermis (Immunohistochemistry with anti-*Treponema pallidum* antibody ×1000, see text for technical details).

The IHC was positive in 24 of 40 skin samples from patients with syphilis and was negative in all 36 samples from non-syphilis controls, yielding a sensitivity of 60% (95% CI 44.8‒75.2), specificity of 100%, positive predictive value of 100%, negative predictive value of 69.2% (95% CI 56.7‒81.8). The accuracy of the test was 78.9% (95% CI 69.8‒88.1). The histopathologic diagnoses proposed in the positive IHC specimens were syphilis (17/24); drug eruption only (4/24); drug eruption and syphilis as an additional hypothesis (2/24); Reiter syndrome (1/24), and no histopathological suggestion was made in 2 samples. Most of the present study cases had spirochetes both in the dermis (Fig. 3A) and in the epidermis (Fig. 3B). Table 1 summarizes the results for IHC.

**Table 1:** Distribution and bacterial load of spirochetes in skin samples positive with anti-*T. pallidum* antibody in the immunohistochemical evaluation.

Location	n (%)
	Total (n = 24)
Epidermis and dermis	18 (75.0)
Dermis (only)	5 (20.8)
Epidermis (only)	1 (4.2)
Epidermal location	19 (79.1)
Basal and Suprabasal	9 (37.5)
Basal (only)	4 (16.6)
Basal, Suprabasal and high strata	3 (12.5)
Suprabasal (only)	2 (8.3)
Basal and high strata	1 (4.2)
Dermal location	23 (95.8)
Extracellular, in the inflammatory infiltrate	21 (87.5)
Dermal vessel wall	20 (83.3)
Intracellular, in inflammatory cells	18 (75.0)
Adnexal epithelium	10 (41.6)
Inside dermal vessel	2 (8.3)
Periadnexal	4 (16.6)
Bacterial Load	
+3	15 (62.5)
+2	3 (12.5)
+1	6 (25.0)

Results of revised clinical data and histopathological findings and their correlations with IHC are presented in Table 2, Table 3. IHC was more commonly positive in samples coming from patients with secondary syphilis (PR = 1.441) or second-tertiary syphilis (PR = 1.286), with anti-HIV non-reagent serology (PR = 1.444) or in immunosuppression by HIV with CD4 less than 200 cells/mm^3^ and VL > 1.000 copies/mL (PR = 1.133). Also, positive results were more prevalent in samples with interface, superficial, and deep perivascular dermatitis (PR = 1.582) or with evident plasma cells ++/++ (PR = 1.547). Samples with superficial and deep perivascular dermatitis (PR = 0.304) or plasma cells +/++ (PR = 0.619) had more negative IHC results. However, these associations were not considered significant (p > 0.05).

**Table 2:** Association between the results of immunohistochemistry with anti-*T. pallidum* antibody and clinical variables of patients with syphilis.

Clinical variable		Skin samples (n = 40) (%)	IHC (n = 40) (%)		PR (95% CI)	p-value^a^
			Positive (n = 24) (60.0)	Negative (n = 16) (40.0)		
Secondary syphilis	Yes	29 (72.5)	19 (47.5)	10 (25.0)	1.441 (0.716‒2.900)	0.213^b^
	No	11 (27.5)	5 (12.5)	6 (15.0)	0.694 (0.345‒1.396)	
Second-tertiary syphilis	Yes	4 (10.0)	3 (7.5)	1 (2.5)	1.286 (0.685‒2.413)	0.471^b^
	No	36 (90.0)	21 (52.5)	15 (37.5)	0.778 (0.414‒1.460)	
Tertiary syphilis	Yes	1 (2.5)	0 (0.0)	1 (2.5)	NA	NA
	No	39 (97.5)	24 (60.0)	15 (37.5)		
Malignant syphilis	Yes	3 (7.5)	1 (2.5)	2 (5.0)	0.536 (0.106‒2.709)	0.348^b^
	No	37 (92.5)	23 (57.5)	14 (35.0)	1.865 (0.369‒9.423)	
Undetermined syphilis	Yes	3 (7.5)	1 (2.5))	2 (5.0)	0.536 (0.106‒2.709)	0.348^b^
	No	37 (92.5)	23 (57.5)	14 (35.0)	1.865 (0.369‒9.423)	
HIV coinfection	Yes	22 (55.0)	11 (27.5)	11 (27.5)	0.692 (0.417‒1.149)	0.154
	No	18 (45.0)	13 (32.5)	5 (12.5)	1.444 (0.870‒2.397)	
CD4 < 200	Yes	7 (17.5)	4 (10.0)	3 (7.5)	0.943 (0.469‒1.895)	0.592^b^
	No	33 (82.5)	20 (50.0)	13 (32.5)	1.061 (0.528‒2.132)	
VL > 1000 copies	Yes	10 (25.0)	6 (15.0)	4 (10.0)	1.000 (0.557‒1.794)	0.64^b^
	No	30 (75.0)	18 (45.0)	12 (30.0)	1.000 (0.557‒1.794)	
CD4 < 200 and VL > 1000 copies	Yes	6 (15.0)	4 (10.0)	2 (5.0)	1.133 (0.602‒2.132)	0.544^b^
	No	34 (85.0)	20 (50.0)	14 (35.0)	0.882 (0.469‒1.660)	

IHC, Immunohistochemistry; VL, HIV viral load; PR, Prevalence Ratio; 95% CI, 95% Confidence Interval; NA, Not Applicable, few cases.

a p-value by Chi-square (χ^2^).

b Fisher’s Exact test.

**Table 3:** Association between immunohistochemistry results with anti-*T. pallidum* antibody and histopathological changes in skin samples from patients with syphilis.

		Skin samples (n = 40) (%)	IHC (n = 40) (%)		PR (95% CI)	p-value^a^
			Positive (n = 24) (60.0)	Negative (n = 16) (40.0)		
**Superficial perivascular tissue reaction patterns only**	Yes	7 (17.5)	3 (7.5)	4 (10.0)	0.673 (0.276‒1.646)	0.273^b^
	No	33 (82.5)	21 (52.5)	12 (30.0)	1.485 (0.608‒3.628)	
Superficial perivascular dermatitis	Yes	1 (2.5)	0	1 (2.5)	NA	NA
	No	39 (97.5)	24 (60.0)	15 (37.5)		
Interface and superficial perivascular dermatitis	Yes	4 (10.0)	2 (5.0)	2 (5.0)	0.818 (0.297‒2.255)	0.529^b^
	No	36 (90.0)	22 (55.0)	14 (35.0)	1.222 (0.443‒3.369)	
Psoriasiform, spongiotic and superficial perivascular dermatitis	Yes	1 (2.5)	1 (2.5)	0	NA	NA
	No	39 (97.5)	23 (57.5)	16 (40.0)		
Interface and psoriasiform dermatitis	Yes	1 (2.5)	0	1 (2.5)	NA	NA
	No	39 (97.5)	24 (60.0)	15 (37.5)		
**Superficial and deep perivascular tissue reaction patterns**	Yes	33 (82.5)	21 (52.5)	12 (30.0)	1.485 (0.608‒3.628)	0.273^b^
	No	7 (17.5)	3 (7.5)	4 (10.0)	0.673 (0.276‒1.646)	
Superficial and deep perivascular dermatitis	Yes	5 (12.5)	1 (2.5)	4 (10.0)	0.304 (0.052‒1.786)	0.073^b^
	No	35 (87.5)	23 (57.5)	12 (30.0)	3.286 (0.560‒19.276)	
Superficial, deep perivascular and periadnexal dermatitis	Yes	3 (7.5)	2 (5.0)	1 (2.5)	1.121 (0.482‒2.606)	0.652^b^
	No	37 (92.5)	22 (55.0)	15 (37.5)	0.892 (0.384‒2.073)	
Interface, superficial and deep perivascular dermatitis	Yes	11 (27.5)	9 (22.5)	2 (5.0)	1.582 (1.010‒2.477)	0.083^b^
	No	29 (72.5)	15 (37.5)	14 (35.0)	0.632 (0.404‒0.990)	
Interface, superficial, deep perivascular and periadnexal dermatitis	Yes	8 (20.0)	5 (12.5)	3 (7.5)	1.053 (0.573‒1.934)	0.601^b^
	No	32 (80.0)	19 (47.5)	13 (32.5)	0.950 (0.517‒1.746)	
Interface and nodular dermatitis	Yes	1 (2.5)	1 (2.5)	0	NA	NA
	No	39 (97.5)	23 (57.5)	16 (40.0)		
Interface, psoriasiform, superficial and deep perivascular dermatitis	Yes	2 (5.0)	0	2 (5.0)	NA	NA
	No	38 (95.0)	24 (60.0)	14 (35.0)		
Interface, psoriasiform, superficial, deep perivascular and periadnexal dermatitis	Yes	2 (5.0)	2 (5.0)	0	NA	NA
	No	38 (95.0)	22 (55.0)	16 (40.0)		
Interface, spongiotic and nodular dermatitis	Yes	1 (2.5)	1 (2.5)	0	NA	NA
	No	39 (97.5)	23 (57.5)	16 (40.0)		
**Melanophages**	Yes	20 (50.0)	11 (27.5)	9 (22.5)	0.845 (0.508‒1.410)	0.519
	No	20 (50.0)	13 (32.5)	7 (17.5)	1.182 (0.709‒1.969)	
**Plasma cells ++/++**	Yes	19 (47.5)	14 (35.0)	5 (12.5)	1.547 (0.917‒2.610)	0.093
	No	21 (52.5)	10 (25.0)	11 (27.5)	0.646 (0.383‒1.090)	
**Plasma cells +/++**	Yes	14 (35.0)	6 (15.0)	8 (20.0)	0.619 (0.321‒1.194)	0.104
	No	26 (65.0)	18 (45.0)	8 (20.0)	1.615 (0.838‒3.116)	
**Plasma cells absent**	Yes	7 (17.5)	4 (10.0)	3 (7.5)	0.943 (0.469‒1.895)	0.592^b^
	No	33 (82.5)	20 (50.0)	13 (32.5)	1.061 (0.528‒2.132)	
**Granulomas**	Yes	17 (42.5)	11 (27.5)	6 (15.0)	1.145 (0.693‒1.891)	0.601
	No	23 (57.5)	13 (32.5)	10 (25.0)	0.874 (0.529‒1.443)	
**Endothelial hyperplasia**	Yes	30 (75.0)	18 (45.0)	12 (30.0)	1.000 (0.557‒1.794)	0.640^b^
	No	10 (25.0)	6 (15.0)	4 (10.0)	1.000 (0.557‒1.794)	

IHC, Immunohistochemistry; (++/++), >30% of inflammatory cell infiltration; (+/++), <30% of inflammatory cell infiltration; PR, Prevalence Ratio; 95% CI, 95% Confidence Interval; NA, Not Applicable, few cases.

a p-value by Chi-square (χ^2^).

b by Fisher Exact test.

## Discussion

A clinical and histopathological description of syphilis cases and a diagnostic accuracy study of immunohistochemistry with anti-*T. pallidum* antibodies were performed. The present study showed a higher frequency of acquired syphilis in males (76.3%) as reported in the literature^1^, ^2^, ^3^, especially with reports among men who have sex with men^1^, ^13^. Syphilis was also observed in adolescents and in older patients, groups commonly considered to be at no or low risk for infection. The secondary phase of the disease was predominant, probably due to the higher frequency of skin biopsies performed at this phase.

Interface dermatitis associated with superficial and deep perivascular dermatitis (27.5%) and superficial, deep perivascular, and periadnexal dermatitis (20%) spotlighted the already reported^14^ mixture of tissue reaction patterns. Endothelial hyperplasia was also frequent (75%), as in previous studies^14^, ^15^. Plasma cells were absent or rare in 7 cutaneous samples (17.5%), in agreement with reports in the literature of scarcity and absence of plasma cells in up to 25% to 33% of the samples^16^, ^17^, ^18^.

Spirochetes and their typical morphology were easily seen with the immunohistochemical method. Similar results are reported with the same^9^, or similar antibodies^6^, ^8^. IHC as a diagnostic test afforded a specific diagnosis of syphilis in 60% of cases, a figure that approaches previous studies (71% to 100% sensitivity)^6^, ^9^. The authors could not ascertain positivity by WS silver staining technique. Quatresooz and Piérard in 2009 showed similar findings with inconclusive or negative results with the WS technique due to the melanin background in the epidermis and reticulin fibrils in the dermis^9^. Other authors, however, found sensitivities of 9% and 60% in skin samples^8^, ^10^.

Spirochetes can predominate in the dermis^6^ or in the epidermis of cutaneous lesions of secondary syphilis^7^, ^8^. Most of the present cases had spirochetes both in the dermis and in the epidermis and a high bacterial load (3+) was predominant. Those results indicate that microorganisms are still diffusively disseminated in the skin during the secondary phase of the disease.

Within the epidermis, spirochetes are mostly located at the basal or supra-basal layer, and in 62.5% of the samples they were not observed at its uppermost part, contrary to the findings by Phelps et al. who rarely observed spirochetes in the lower portions of the epidermis^8^. The finding of spirochetes in the follicular epithelium and around adnexa in 58.3% of positive samples further supports the microorganism epitheliotropism theory^7^.

Vascular changes are an important component of inflammatory tissue response in syphilis. Endothelial hyperplasia was a common and evident finding and numerous immunostained spirochetes were detected within the vessel wall. Martin-Ezquerra et al. also found a vasculotropic pattern, but, mostly in lesions of primary syphilis^7^.

No significant correlation between IHC and clinical or histopathological characteristics was observed. Nevertheless, the prevalence of positive IHC results was higher in samples from patients without HIV coinfection (PR = 1.444) and in those with immunosuppression by HIV with CD4 less than 200 cells/mm^3^ and VL > 1.000 copies/mL (PR = 1.133). These results suggest that HIV coinfection *per se* had no main impact on spirochete detection, but the immunosuppression state might influence spirochete spread in syphilis samples. Similar to the present findings, previous research has reported high bacterial load in skin samples from patients with a CD4 count below 250 cells^10^ or in immunosuppression states associated with early malignant syphilis^11^.

Syphilis skin samples with interface, superficial and deep perivascular dermatitis, and with visible plasma cells in the infiltrate (++/++) showed a higher prevalence of positive results in IHC compared to samples without these findings (PR = 1.582, PR = 1.547 respectively). On the contrary, samples with superficial and deep perivascular dermatitis (PR = 0.304) or few plasma cells (+/++) (PR = 0.619) suggest that even with negative IHC results, a diagnosis of syphilis must not be excluded.

## Conclusion

Antigen detection in an immunohistochemical protocol highlighted *Treponema pallidum* spirochetes and enabled an undoubted diagnosis of syphilis on tissue samples. WS staining was considered inefficient in bacterial detection and offered no contribution to a specific diagnosis of the disease.

## Ethics committees

The ethics committees of Clementino Fraga Filho University Hospital (HUCFF) of the Federal University of Rio de Janeiro (UFRJ) and Evandro Chagas National Institute of Infectious Diseases (INI), Oswaldo Cruz Foundation (Fiocruz), Brazil, approved the study. The committees waived informed consent because no personally identifiable information was included in the data set used for analysis.

## Financial support

None declared.

## Authors' contributions

Mariana Freitas de Assis Pereira Rosa: Conception and design of the study; data collection, or data analysis and interpretation; statistical analysis; article writing or critical review of important intellectual content; obtaining, analyzing, and interpreting data; effective participation in research orientation; critical review of the literature; approval of the final version of the manuscript.

Leonardo Pereira Quintella: Conception and design of the study; data collection, or data analysis and interpretation; statistical analysis; article writing or critical review of important intellectual content; obtaining, analyzing, and interpreting data; effective participation in research orientation; critical review of the literature; approval of the final version of the manuscript.

Luiz Claudio Ferreira: Data collection; obtaining, analyzing, and interpreting data.

Tullia Cuzzi: Conception and design of the study; data collection, or data analysis and interpretation; statistical analysis; article writing or critical review of important intellectual content; obtaining, analyzing, and interpreting data; effective participation in research orientation; critical review of the literature; approval of the final version of the manuscript.

## Conflicts of interest

None declared.
